# Pediatric HIV Case Identification Across 22 PEPFAR-Supported Countries During the COVID-19 Pandemic, October 2019–September 2020

**DOI:** 10.15585/mmwr.mm7128a2

**Published:** 2022-07-15

**Authors:** Ariana M. Traub, Amy Medley, Jessica Gross, Margo Sloan, Anouk Amzel, Megan M. Gleason, Nimasha B. Fernando, Vincent Wong, Michael P. Grillo, Hilary T. Wolf, Teeb Al-Samarrai, Alean Frawley, Mosarwa Segwabe, Catherine Motswere, Evelyne Baramperanye, Valery Nzima, Magdalene Mange Mayer, Shirish Balachandra, Francois-Xavier N'siesi, Henri O. Longuma, Patricia Nyembo, Sikhathele Mazibuko, Tsegaye Tilahun, Wondimu Teferi, Olbeg Desinor, Jean-Louis Reginald, Teresa Simiyu, Lennah Nyabiage, Justine Mirembe, Mamorapeli Ts’oeu, Gerald Zomba, Mtemwa Nyangulu, Argentina Wate, Jessica Greenberg Cowan, Denis Mali, Ismelda Pietersen, Dolapo Ogundehin, Dennis Onotu, Akudo Ikpeazu, Esron Niyonsaba, Jackson Bamwesigye, Hlamalani Mabasa, Gurpreet Kindra, Sudhir Bunga, Fredrick Rwegerera, Edward Machage, Galal King'ori, Jacqueline Calnan, Esther Nazziwa, Godfrey Lingenda, Kebby Musokotwane, Ruth Bulaya-Tembo, Talent Maphosa, Meena Srivastava

**Affiliations:** ^1^Office of HIV/AIDS, U.S. Agency for International Development, Washington, D.C.; ^2^Division of Global HIV & TB, Center for Global Health, CDC; ^3^U.S. Department of Defense HIV/AIDS Prevention Program, Defense Health Agency, San Diego, California; ^4^Office of the Global AIDS Coordinator and Health Diplomacy, U.S. Department of State, Washington, D.C.; ^5^Division of Global HIV & TB, Center for Global Health, CDC Angola; ^6^U.S. Agency for International Development, Botswana; ^7^Division of Global HIV & TB, Center for Global Health, CDC Botswana; ^8^U.S. Agency for International Development, Burundi; ^9^U.S. Agency for International Development, Cameroon; ^10^Division of Global HIV & TB, Center for Global Health, CDC Cameroon; ^11^Division of Global HIV & TB, Center for Global Health, CDC Côte d’Ivoire; ^12^U.S. Agency for International Development, Democratic Republic of the Congo; ^13^Division of Global HIV & TB, Center for Global Health, CDC Democratic Republic of the Congo; ^14^National AIDS Control Committee, Democratic Republic of the Congo; ^15^Division of Global HIV & TB, Center for Global Health, CDC Eswatini; ^16^U.S. Agency for International Development, Ethiopia; ^17^Division of Global HIV & TB, Center for Global Health, CDC Ethiopia; ^18^U.S. Agency for International Development, Haiti; ^19^Division of Global HIV & TB, Center for Global Health, CDC Haiti; ^20^U.S. Agency for International Development, Kenya; ^21^Division of Global HIV & TB, Center for Global Health, CDC Kenya; ^22^U.S. Agency for International Development, Lesotho; ^23^Division of Global HIV & TB, Center for Global Health, CDC Lesotho; ^24^U.S. Agency for International Development, Malawi; ^25^Division of Global HIV & TB, Center for Global Health, CDC Malawi; ^26^U.S. Agency for International Development, Mozambique; ^27^Division of Global HIV & TB, Center for Global Health, CDC Mozambique; ^28^U.S. Agency for International Development, Namibia; ^29^Division of Global HIV & TB, Center for Global Health, CDC Namibia; ^30^U.S. Agency for International Development, Nigeria; ^31^Division of Global HIV & TB, Center for Global Health, CDC Nigeria; ^32^Federal Ministry of Health, Nigeria; ^33^U.S. Agency for International Development, Rwanda; ^34^Division of Global HIV & TB, Center for Global Health, CDC Rwanda; ^35^U.S. Agency for International Development, South Africa; ^36^Division of Global HIV & TB, Center for Global Health, CDC South Africa; ^37^Division of Global HIV & TB, Center for Global Health, CDC South Sudan; ^38^U.S. Agency for International Development, Tanzania; ^39^Division of Global HIV & TB, Center for Global Health, CDC Tanzania; ^40^U.S. Agency for International Development, Uganda; ^41^Division of Global HIV & TB, Center for Global Health, CDC Uganda; ^42^U.S. Agency for International Development, Zambia; ^43^Division of Global HIV & TB, Center for Global Health, CDC Zambia; ^44^U.S. Agency for International Development, Zimbabwe; ^45^Division of Global HIV & TB, Center for Global Health, CDC Zimbabwe.

During 2020, an estimated 150,000 persons aged 0–14 years acquired HIV globally (*1*). Case identification is the first step to ensure children living with HIV are linked to life-saving treatment, achieve viral suppression, and live long, healthy lives. Successful interventions to optimize pediatric HIV testing during the COVID-19 pandemic are needed to sustain progress toward achieving Joint United Nations Programme on HIV/AIDS (UNAIDS) 95–95–95 targets.* Changes in HIV testing and diagnoses among persons aged 1–14 years (children) were assessed in 22 U.S. President’s Emergency Plan for AIDS Relief (PEPFAR)-supported countries during October 1, 2019–September 30, 2020. This period corresponds to the two fiscal quarters before the COVID-19 pandemic (i.e., Q1 and Q2) and the two quarters after the pandemic began (i.e., Q3 and Q4). Testing was disaggregated by age group, testing strategy, and fiscal year quarter. During October 2019–September 2020, PEPFAR supported 4,312,343 HIV tests and identified 74,658 children living with HIV (CLHIV). The number of HIV tests performed was similar during Q1 and Q2, decreased 40.1% from Q2 to Q3, and increased 19.7% from Q3 to Q4. The number of HIV cases identified among children aged 1–14 years (cases identified) increased 7.4% from Q1 to Q2, decreased 29.4% from Q2 to Q3, and increased 3.3% from Q3 to Q4. Although testing in outpatient departments decreased 21% from Q1 to Q4, testing from other strategies increased during the same period, including mobile testing by 38%, facility-based index testing (offering an HIV test to partners and biological children of persons living with HIV) by 8%, and testing children with signs or symptoms of malnutrition within health facilities by 7%. In addition, most tests (61.3%) and cases identified (60.9%) were among children aged 5–14 years (school-aged children), highlighting the need to continue offering HIV testing to older children. These findings provide important information on the most effective strategies for identifying CLHIV during the COVID-19 pandemic. HIV testing programs should continue to use programmatic, surveillance, and financial data at both national and subnational levels to determine the optimal mix of testing strategies to minimize disruptions in pediatric case identification during the COVID-19 pandemic.

Monitoring, evaluation, and reporting indicators^†^ from 22 of 50 PEPFAR-supported countries were analyzed to assess changes in the number of HIV tests conducted and the number of cases identified among children during the two fiscal quarters before the start of the COVID-19 pandemic (October 2019–March 2020) and the two fiscal quarters after the pandemic began (April–September 2020). These 22 countries were selected because they account for >80% of CLHIV not receiving HIV treatment globally. Percent positivity was calculated by dividing the number of positive test results by the total number of tests reported. HIV test outcomes are reported overall, and by country, age group, testing strategy, and fiscal year quarter. Testing strategies include provider-initiated testing and counseling (PITC) in outpatient departments, tuberculosis clinics, malnutrition services, well-child clinics (for infants and children aged <5 years), and inpatient wards; index testing in facility and community settings; voluntary counseling and testing (VCT) initiated by clients; and mobile testing in the community. This protocol was reviewed in accordance with CDC human research protection procedures, determined to be a non-research public health program activity, and conducted consistent with applicable federal law and CDC policy.^§^

Of the 4,312,343 HIV tests conducted among children in the 22 countries, approximately one quarter (22.6%) occurred in South Africa. Among the 74,658 cases identified (representing an overall 1.7% positivity rate), approximately one half (54.7%) were in Mozambique, Nigeria, South Africa, and Tanzania, with Mozambique identifying the most cases (12,367; 16.6%) (Table 1). The majority of tests conducted (61.3%) and of cases identified (60.9%) were among school-aged children (Table 1). Percent positivity was highest among children aged 5–9 years (2.1%) followed by those aged 1–4 (1.8%) and 10–14 (1.5%) years.

**TABLE 1:** Number of tests and number and percentage of children and adolescents aged 1–14 years identified with HIV, by age group — 22 PEPFAR-supported countries, October 2019–September 2020

Country	No. of HIV tests conducted (%)				No. of HIV-positive tests (%)			
	All	1–4 yrs	5–9 yrs	10–14 yrs	All	1–4 yrs	5–9 yrs	10–14 yrs
Angola	6,949	2,874 (41.4)	2,281 (32.8)	1,794 (25.8)	381 (5.5)	174 (6.1)	129 (5.7)	78 (4.3)
Botswana	4,524	1,556 (34.4)	367 (8.1)	2,601 (57.5)	43 (1.0)	15 (1.0)	16 (4.4)	12 (0.5)
Burundi	23,349	13,392 (57.4)	5,130 (22.0)	4,827 (20.7)	414 (1.8)	143 (1.1)	143 (2.8)	128 (2.7)
Cameroon	104,328	39,852 (38.2)	32,773 (31.4)	31,703 (30.4)	1,906 (1.8)	840 (2.1)	639 (1.9)	427 (1.3)
Côte d’Ivoire	117,773	47,254 (40.1)	34,567 (29.4)	35,952 (30.5)	1,148 (1.0)	538 (1.1)	316 (0.9)	294 (0.8)
DRC	98,410	35,296 (35.9)	32,888 (33.4)	30,226 (30.7)	4,087 (4.2)	1,637 (4.6)	1,454 (4.4)	996 (3.3)
Eswatini	27,618	8,028 (29.1)	6,864 (24.9)	12,726 (46.1)	449 (1.6)	116 (1.4)	112 (1.6)	221 (1.7)
Ethiopia	354,066	230,396 (65.1)	51,330 (14.5)	72,340 (20.4)	1,451 (0.4)	619 (0.3)	394 (0.8)	438 (0.6)
Haiti	33,772	16,321 (48.3)	8,130 (24.1)	9,321 (27.6)	452 (1.3)	185 (1.1)	124 (1.5)	143 (1.5)
Kenya	297,984	79,364 (26.6)	85,125 (28.6)	133,495 (44.8)	4,693(1.6)	1,803 (2.3)	1,515 (1.8)	1,375 (1.0)
Lesotho	61,645	19,731 (32.0)	16,105 (26.1)	25,809 (41.9)	226 (0.4)	83 (0.4)	45 (0.3)	98 (0.4)
Malawi	155,859	73,200 (47.0)	30,423 (19.5)	52,236 (33.5)	3,028 (1.9)	1,300 (1.8)	711 (2.3)	1,017 (1.9)
Mozambique	637,575	222,439 (34.9)	201,503 (31.6)	213,633 (33.5)	12,367 (1.9)	4,753 (2.1)	4,524 (2.2)	3,090 (1.4)
Namibia	12,268	5,245 (42.8)	3,398 (27.7)	3,625 (29.5)	251 (2.0)	100 (1.9)	78 (2.3)	73 (2.0)
Nigeria	405,589	122,720 (30.3)	114,727 (28.3)	168,142 (41.5)	9,471 (2.3)	3,817 (3.1)	2,785 (2.4)	2,869 (1.7)
Rwanda	8,963	1,957 (21.8)	3,452 (38.5)	3,554 (39.7)	147 (1.6)	70 (3.6)	49 (1.4)	28 (0.8)
South Africa	972,761	441,881 (45.4)	211,183 (21.7)	319,697(32.9)	10,726 (1.1)	3,561 (0.8)	2,638 (1.2)	4,527 (1.4)
South Sudan	36,577	14,554 (39.8)	8,953 (24.5)	13,070 (35.7)	475 (1.3)	274 (1.9)	101 (1.1)	100 (0.8)
Tanzania	236,162	89,164 (37.8)	74,053 (31.4)	72,945 (30.9)	8,282 (3.5)	3,370 (3.8)	2,684 (3.6)	2,228 (3.1)
Uganda	354,014	81,255 (23.6)	81,065 (23.5)	182,694 (53.0)	5,031 (1.5)	2,089 (2.6)	1,542 (1.9)	1,400 (0.8)
Zambia	244,555	89,148 (36.5)	64,176 (26.2)	91,231 (37.3)	7,153 (2.9)	2,835 (3.2)	2,104 (3.3)	2,214 (2.4)
Zimbabwe	126,602	32,891 (26.0)	14,663 (11.6)	79,048 (62.4)	2,477 (2.0)	893 (2.7)	665 (4.5)	919 (1.2)
**Total**	**4,312,343**	**1,668,518 (38.7)**	**1,083,156 (25.1)**	**1,560,669 (36.2)**	**74,658 (1.7)**	**29,215 (1.8)**	**22,768 (2.1)**	**22,675 (1.5)**

**Source:** PEPFAR Monitoring, Evaluation, and Reporting Database, Data for Accountability, Transparency, and Impact Monitoring database, October 2019–September 2020.

**Abbreviations:** DRC = Democratic Republic of the Congo; PEPFAR = U.S. President’s Emergency Plan for AIDS Relief.

The number of HIV tests conducted among children decreased 40.1% from Q2 to Q3 across all 22 countries at the start of the COVID-19 pandemic but increased 19.7% from Q3 to Q4 as programs began making shifts in their HIV testing strategies. Similarly, the number of cases identified decreased 29.4% from Q2 to Q3 but increased 3.3% from Q3 to Q4. Seventeen of the 22 countries reported that the number of cases identified increased from Q3 to Q4. By Q4, case identification had surpassed pre–COVID-19 levels in six countries, (Q4:Q1 ratio ≥1.0), returned to pre–COVID-19 levels in three countries (Q4:Q1 ratio >0.95–<1.0), and remained below pre–COVID-19 levels in 13 countries (Q4:Q1 ratio <0.95) (Table 2).

**TABLE 2:** Number of tests and number and percentage of children and adolescents aged 1–14 years identified with HIV, by quarter — 22 PEPFAR-supported countries, October 2019–September 2020

Country	No. of HIV tests conducted						No. of HIV-positive tests (%)					
	All	Oct–Dec 2019	Jan–Mar 2020	Apr–Jun 2020	Jul–Sep 2020	Ratio Q4 versus Q1*	All	Oct–Dec 2019	Jan–Mar 2020	Apr–Jun 2020	Jul–Sep 2020	Ratio Q4 versus Q1*
Angola	6,949	1,477	2,173	1,336	1,963	1.33	381 (5.5)	86 (5.8)	101 (4.6)	68 (5.1)	126 (6.4)	1.47
Botswana	4,524	3,376	667	209	272	0.08	43 (1.0)	16 (0.5)	11 (1.6)	10 (4.8)	6 (2.2)	0.38
Burundi	23,349	5,817	5,642	6,452	5,438	0.93	414 (1.8)	100 (1.7)	106 (1.9)	123 (1.9)	85 (1.6)	0.85
Cameroon	104,336	20,320	22,297	29,540	32,179	1.58	1,905 (1.8)	319 (1.6)	505 (2.3)	534 (1.8)	547 (1.7)	1.71
Côte d’Ivoire	117,773	34,491	32,157	26,602	24,523	0.71	1,148 (1.0)	284 (0.8)	302 (0.9)	259 (1.0)	303 (1.2)	1.07
DRC	98,410	22,811	27,226	23,610	24,763	1.09	4,087 (4.2)	894 (3.9)	1,096 (4.0)	1,001 (4.2)	1,096 (4.4)	1.23
Eswatini	27,618	5,489	7,971	2,839	11,319	2.06	449 (1.6)	110 (2.0)	144 (1.8)	72 (2.5)	123 (1.1)	1.12
Ethiopia	354,075	135,267	108,439	50,760	59,609	0.44	1,451 (0.4)	535 (0.4)	436 (0.4)	228 (0.4)	252 (0.4)	0.47
Haiti	33,772	9,038	10,469	5,024	9,241	1.02	452 (1.3)	107 (1.2)	133 (1.3)	78 (1.6)	134 (1.5)	1.25
Kenya	297,985	94,057	79,212	57,048	67,668	0.72	4,689 (1.6)	1,246 (1.3)	1,440 (1.8)	971 (1.7)	1,032 (1.5)	0.83
Lesotho	61,645	30,726	22,387	4,803	3,729	0.12	226 (0.4)	79 (0.3)	88 (0.4)	34 (0.7)	25 (0.7)	0.32
Malawi	155,859	62,284	40,349	21,721	31,505	0.51	3,028 (1.9)	1,062 (1.7)	728 (1.8)	514 (2.4)	724 (2.3)	0.68
Mozambique	637,570	173,106	201,520	125,967	136,977	0.79	12,367 (1.9)	3,096 (1.8)	3,875 (1.9)	2,626 (2.1)	2,770 (2.0)	0.89
Namibia	12,268	3,236	3,749	3,006	2,277	0.70	251 (2.0)	66 (2.0)	67 (1.8)	68 (2.3)	50 (2.2)	0.76
Nigeria	405,589	94,283	104,003	83,941	123,362	1.31	9,471 (2.3)	2,489 (2.6)	2,386 (2.3)	2,166 (2.6)	2,430 (2.0)	0.98
Rwanda	8,963	2,159	2,233	2,207	2,364	1.09	147 (1.6)	46 (2.1)	34 (1.5)	33 (1.5)	34 (1.4)	0.74
South Africa	972,760	265,547	318,778	168,309	220,126	0.83	10,726 (1.1)	3,514 (1.3)	3,682 (1.2)	1,728 (1.0)	1,802 (0.8)	0.51
South Sudan	36,577	11,430	9,529	7,464	8,154	0.71	475 (1.3)	112 (1.0)	160 (1.7)	93 (1.2)	110 (1.3)	0.98
Tanzania	236,162	58,561	71,216	59,803	46,582	0.80	8,282 (3.5)	2,356 (4.0)	2,411 (3.4)	2,050 (3.4)	1,465 (3.1)	0.62
Uganda	345,016	102,383	118,751	49,706	74,176	0.72	5,031 (1.5)	1,155 (1.1)	1,769 (1.5)	982 (2.0)	1,125 (1.5)	0.97
Zambia	244,555	81,290	72,208	47,150	43,907	0.54	7,153 (2.9)	2,159 (2.7)	1,863 (2.6)	1,652 (3.5)	1,479 (3.4)	0.69
Zimbabwe	126,602	51,328	52,754	10,051	12,469	0.24	2,479 (2.0)	828 (1.6)	844 (1.6)	362 (3.6)	445 (3.6)	0.54
**Total**	**4,312,357**	**1,268,476**	**1,313,730**	**787,548**	**942,603**	**0.74**	**74,655 (1.7)**	**20,659 (1.6)**	**22,181 (1.7)**	**15,652 (2.0)**	**16,163 (1.7)**	**0.78**

**Source:** PEPFAR Monitoring, Evaluation, and Reporting Database, Data for Accountability, Transparency, and Impact Monitoring database, October 2019–September 2020.

**Abbreviations:** DRC = Democratic Republic of the Congo; PEPFAR = U.S. President’s Emergency Plan for AIDS Relief; Q1 = quarter 1; Q4 = quarter 4.

* Q1 (Oct–Dec 2019) and Q4 (Jul–Sep 2020).

Approximately one half (47.9%) of HIV tests were conducted in outpatient department settings, followed by facility-based index testing (12.1%), well-child clinics (8.1%), and mobile testing (5.4%). PITC in outpatient departments identified the largest number of cases (24,812; 33.2%), followed by facility-based index testing (24,372; 32.6%), community-based index testing (5,922; 7.9%), and VCT (5,034; 6.7%). Similarly, the percent positivity was highest for PITC in tuberculosis clinics (5.4%), followed by facility-based index testing (4.6%), community-based index testing (3.6%), and VCT (2.6%). Facility and community-based index testing, combined, identified the most cases across the four quarters (40.5%; positivity rate = 4.4%), despite only representing 18.3% of all testing.

By Q4, the number of tests conducted returned to pre–COVID-19 levels (Q4:Q1 ratio >0.95) for three strategies: mobile testing, facility-based index testing, and PITC among malnourished children (Table 3). However, the number of tests conducted was <75% of pre–COVID-19 levels in Q4 for PITC in inpatient wards and well-child clinics, VCT, and index testing in community settings. The number of cases identified decreased from Q2 to Q3 across all strategies except inpatient wards, where the number increased by 28.8% and the percentage of HIV-positive test results nearly doubled from 1.2% to 2.2%. By Q4, case identification only reached pre–COVID-19 levels for facility-based index testing and PITC among malnourished children.

**TABLE 3:** Number of children and adolescents aged 1–14 years receiving testing, identified as HIV-positive, and percent positivity by HIV testing strategy — 22 PEPFAR-supported countries, October 2019–September 2020

HIV testing strategy	No. of HIV tests conducted (%)						No. of HIV-positive tests (%)						
	All	Oct–Dec 2019	Jan–Mar 2020	Apr–Jun 2020	Jul–Sep 2020	Ratio Q4 versus Q1*	All	Percent positivity^†^	Oct–Dec 2019	Jan–Mar 2020	Apr–Jun 2020	Jul–Sep 2020	Ratio Q4 versus Q1*
Outpatient department	2,065,526 (47.9)	585,548 (46.2)	616,578 (46.9)	402,996 (51.2)	460,404 (48.8)	0.79	24,812 (33.2)	1.2	6,625 (1.1)	7,808 (1.3)	5,039 (1.3)	5,340 (1.2)	0.81
Index (facility)	523,931 (12.1)	127,743 (10.1)	140,453 (10.7)	117,241 (14.9)	138,494 (14.7)	1.08	24,327 (32.6)	4.6	6,154 (4.8)	7,000 (5.0)	5,491 (4.7)	5,682 (4.1)	0.92
Index (community)	162,966 (3.8)	47,751 (3.8)	62,559 (4.8)	19,950 (2.5)	32,706 (3.5)	0.68	5,922 (7.9)	3.6	1,806 (3.8)	1,956 (3.1)	950 (4.8)	1,210 (3.7)	0.67
Inpatient wards	168,420 (3.9)	49,425 (3.9)	49,144 (3.7)	33,924 (4.3)	35,927 (3.8)	0.73	2,337 (3.1)	1.4	582 (1.2)	590 (1.2)	760 (2.2)	405 (1.1)	0.70
Tuberculosis clinics	39,378 (0.9)	10,128 (0.8)	12,073 (0.9)	8,147 (1.0)	9,030 (1.0)	0.89	2,124 (2.8)	5.4	674 (6.7)	618 (5.1)	470 (5.8)	362 (4.0)	0.54
Malnutrition clinics	27,513 (0.6)	6,406 (0.5)	7,287 (0.6)	6,986 (0.9)	6,834 (0.7)	1.07	284 (0.4)	1.0	75 (1.2)	77 (1.1)	59 (0.8)	73 (1.1)	0.97
VCT	192,269 (4.5)	67,926 (5.4)	50,780 (3.9)	32,189 (4.1)	41,374 (4.4)	0.61	5,034 (6.7)	2.6	1,578 (2.3)	1,504 (3.0)	960 (3.0)	992 (2.4)	0.63
Well-child clinics^§^	349,315 (8.1)	112,522 (8.9)	104,654 (8.0)	62,483 (7.9)	69,656 (7.4)	0.62	2,591 (3.5)	0.7	858 (0.8)	664 (0.6)	602 (1.0)	467 (0.7)	0.54
Mobile	233,476 (5.4)	49,386 (3.9)	74,792 (5.7)	41,168 (5.2)	68,130 (7.2)	1.38	3,015 (4.0)	1.3	835 (1.7)	885 (1.2)	597 (1.5)	698 (1.0)	0.84
All other strategies	549,563 (12.7)	211,641 (16.7)	195,410 (14.9)	62,464 (7.9)	80,048 (8.5)	0.38	4,209 (5.6)	0.8	1,472 (0.7)	1,079 (0.6)	724 (1.2)	934 (1.2)	0.63
**Total**	**4,312,357**	**1,268,476**	**1,313,730**	**787,548**	**942,603**	**0.74**	**74,655**	**1.7**	**20,659 (1.6)**	**22,181 (1.7)**	**15,652 (2.0)**	**16,163 (1.7)**	**0.78**

**Source:** PEPFAR Monitoring, Evaluation, and Reporting Database, Data for Accountability, Transparency, and Impact Monitoring database, October 2019–September 2020.

**Abbreviations:** PEPFAR = U.S. President’s Emergency Plan for AIDS Relief; Q1 = quarter 1; Q4 = quarter 4; VCT = voluntary counseling and testing.

* Ratio of the number of tests conducted and positive test results received comparing Q1 (Oct–Dec 2019) and Q4 (Jul–Sep 2020).

^† ^Number of HIV-positive test results divided by the total number of tests conducted.

^§^ For infants and children aged <5 years.

## Discussion

Findings from this report suggest progress toward reaching the UNAIDS 95–95–95 targets for CLHIV were negatively affected during the COVID-19 pandemic, especially during April–June 2020. Although the number of HIV tests conducted and cases identified increased from Q3 to Q4, the overall number of children diagnosed with HIV during Q4 remained below pre–COVID-19 levels. Although more resource intensive (*2*), index testing remains a priority for identifying children before they develop advanced disease, and during the COVID-19 pandemic when children and caregivers are less likely to seek outpatient services (*3*). Prioritizing the identification and testing of the biological children of key populations (i.e., persons who engage in sex work, men who have sex with men, persons who inject drugs, persons who identify as transgender, and persons who are incarcerated in prisons and other closed settings) living with HIV is also critical given their increased risk and vulnerabilities (*4*).

Although community index testing and mobile testing did not identify as many cases as did PITC, they also remain important strategies to identify children unable to access health care (*5*), and to limit potential exposures at health care facilities during the COVID-19 pandemic. In a similar recent analysis of 16 countries, those countries that maintained or increased community-based testing, including index testing, were able to mitigate declines in the number of cases identified during the COVID-19 pandemic (*5*). In the current analysis, the number of tests conducted and cases identified in community-based index testing during Q4 did not reach pre–COVID-19 levels, although this strategy did have a relatively high percent positivity in Q4. Community-based testing strategies are often more expensive than facility-based approaches (*6*). Therefore, each country program will have to determine the cost-benefit ratio of different testing strategies using national and subnational data to guide decisions on which strategies to implement for pediatric case finding. Orphans and vulnerable children programs, which are integral to community-based care for CLHIV, can also provide support to facilitate HIV testing (*7*). Programs might consider accelerating policies allowing the distribution of oral self-test kits to caregivers to screen their biological children aged ≥2 years for HIV to reduce barriers to HIV testing, decrease visits to health care facilities, and close gaps in elicitation and testing of biological contacts (*8*).

Children infected through perinatal transmission might be seen at health care facilities (e.g., tuberculosis clinics, malnutrition clinics, and inpatient wards) with advanced disease if they are not diagnosed through other testing strategies. In this analysis, the percent positivity was highest for PITC in tuberculosis clinics; testing in inpatient wards was the only strategy that had an increase in cases identified during the first quarter of the COVID-19 pandemic. Universal testing at these entry points is therefore crucial, particularly during the COVID-19 pandemic when children might seek care with more advanced disease. In addition, most tests conducted (61.3%) and cases identified (60.9%) were among school-aged children. This finding highlights the ongoing need for both early infant diagnosis to identify and link children to treatment at an earlier age and HIV testing services among older children because studies indicate children infected during breastfeeding can survive into adolescence even without treatment (*9*).

The findings in this report are subject to at least four limitations. First, although countries follow PEPFAR monitoring and reporting guidance, data quality and reporting by testing strategy vary across countries. This caveat is particularly true for community-based testing in which contacts of known persons living with HIV might not always be accurately reflected under index testing. Second, PEPFAR indicators monitor the number of tests conducted, not the number of persons tested. Thus, the number of tests conducted and HIV-positive test results returned might be higher than the number of persons who received testing. Third, the impacts, restrictions, and adaptations to the COVID-19 pandemic varied across countries. This analysis cannot fully account for the impact of these variations on the results presented. Further qualitative assessments might provide a more in-depth understanding of how COVID-19 affected the provision and uptake of HIV testing across multiple waves of the pandemic. Finally, some countries did not use all the testing strategies included in this analysis.

Case identification is the first step to ensure CLHIV are linked to life-saving treatment, achieve viral suppression, and live long, healthy lives. During the COVID-19 pandemic, many PEPFAR-supported countries experienced disruptions in case identification among CLHIV. Six countries (Angola, Cameroon, Côte d’Ivoire, Democratic Republic of Congo, Eswatini, and Haiti), however, were able to exceed pre–COVID-19 case identification levels, using a combination of high yield strategies, including facility index testing, mobile testing, and testing children with signs or symptoms of malnutrition. These findings provide important information for countries and programs on the most effective strategies for identifying CLHIV during the COVID-19 pandemic. HIV testing programs should continue to use programmatic, surveillance, and financial data at both national and subnational levels to determine the optimal mix of testing strategies to minimize disruptions in pediatric case identification during the COVID-19 pandemic surges and other public health crises.

**Summary**

**What is already known about this topic?**

Identifying and linking children living with HIV to treatment is essential to reduce morbidity and mortality.

**What is added by this report?**

During the first 3 months of the COVID-19 pandemic, HIV testing and case identification among children and adolescents aged 1–14 years in 22 PEPFAR-supported countries decreased by 40.1% and 29.4%, respectively. Although outpatient testing decreased (21%), testing increased for other strategies, including mobile (38%), facility-based index (8%), and malnutrition (7%), suggesting these strategies can mitigate the impact of COVID-19 on pediatric case identification.

**What are the implications for public health practice?**

HIV testing programs can use programmatic, surveillance, and financial data to determine the optimal mix of testing strategies during the COVID-19 pandemic.

## Footnotes

* The UNAIDS 95–95–95 strategy to reach HIV epidemic control by 2030 calls for 95% of all persons living with HIV (PLHIV) to know their status; 95% of diagnosed PLHIV to be receiving antiretroviral treatment; and 95% of PLHIV receiving antiretroviral treatment to be virally suppressed (defined as <200 copies of HIV per mL of blood).

† Monitoring, evaluation, and reporting indicators: number of persons who received HIV testing services and their test results and number of persons receiving positive test results for HIV.

§ 45 C.F.R. part 46, 21 C.F.R. part 56; 42 U.S.C. Sect. 241(d); 5 U.S.C. Sect. 552a; 44 U.S.C. Sect. 3501 et seq.
